# Progress towards achieving and maintaining maternal and neonatal tetanus elimination in the African region

**DOI:** 10.11604/pamj.supp.2017.27.3.11783

**Published:** 2017-06-22

**Authors:** Alison Delano Ridpath, Heather Melissa Scobie, Messeret Eshetu Shibeshi, Ahmadu Yakubu, Flint Zulu, Azhar Abid Raza, Balcha Masresha, Rania Tohme

**Affiliations:** 1 Centers for Disease Control and Prevention, Global Immunization Division, Atlanta, GA, USA; 2 World Health Organization, Intercountry Support Team for East and Southern Africa, Harare, Zimbabwe; 3 World Health Organization, Family, Women and Children’s Health Cluster, Geneva, Switzerland; 4 United Nations Children’s Fund, Health Section, Program Division, New York, NY, USA

**Keywords:** Africa, tetanus, immunization

## Abstract

Despite the availability of effective tetanus prevention strategies, as of 2016, Maternal and Neonatal Tetanus Elimination (MNTE) has not yet been achieved in 18 countries globally. In this paper, we review the status of MNTE in the World Health Organization African Region (AFR),and provide recommendations for achieving and maintaining MNTE in AFR. As of November 2016, 37 (79%) AFR countries have achieved MNTE, with 10 (21%) countries remaining. DTP3 coverage increased from 52% in 2000 to 76% in 2015. In 2015, coverage with at least 2 doses of tetanus containing vaccine (TT2+) and proportion of newborns protected at birth (PAB) were 69% and 77%, compared with 44% and 62% in 2000, respectively. Since 1999, over 79 million women of reproductive age (WRA) have been vaccinated with TT2+ through supplementary immunization activities (SIAs). Despite the progress, only 54% of births were attended by skilled birth attendants (SBAs), 5 (11%) countries provided the 3 WHO-recommended booster doses to both sexes, and about 5.5 million WRA still need to be reached with SIAs. Coverage disparities still exist between countries that have achieved MNTE and those that have not. In 2015, coverage with DTP3 and PAB were higher in MNTE countries compared with those yet to achieve MNTE: 84% vs. 68% and 86% vs. 69%, respectively. Challenges to achieving MNTE in the remaining AFR countries include weak health systems, competing priorities, insufficient funding, insecurity, and sub-optimal neonatal tetanus (NT) surveillance. To achieve and maintain MNTE in AFR, increasing SBAs and tetanus vaccination coverage, integrating tetanus vaccination with other opportunities (e.g., polio and measles campaigns, mother and child health days), and providing appropriately spaced booster doses are needed. Strengthening NT surveillance and conducting serosurveys would ensure appropriate targeting of MNTE activities and high-quality information for validating the achievement and maintenance of elimination.

## Introduction

Tetanus is a non-communicable vaccine-preventable disease caused by the bacterium *Clostridium tetani (C. tetani)*. The disease cannot be eradicated because C. tetani spores exist in the environment [1]. Although tetanus can occur in all age-groups, neonates and women with recent pregnancies are most at risk, particularly when childbirths (or terminations of pregnancies) take place under unhygienic conditions [2]. Neonatal tetanus (NT) occurs during the first 28 days of life and maternal tetanus occurs during or within the first 6 weeks after pregnancy. Case-fatality rates from tetanus in resource-limited settings can be up to 100%, though with adequate medical care rates can be reduced to 10-20% [3].

Tetanus toxoid containing vaccine (TTCV) is one of the most effective, safe, heat-stable, and inexpensive vaccines ever developed [4]. TTCV can be given safely during pregnancy to protect both mothers and newborns [5]. Vaccines containing tetanus for infants and pregnant women have been part of the WHO Expanded Program on Immunizations since its inception. WHO recommends a 6 dose schedule of TTCV including 3 doses in infancy and 3 booster doses, 1 each during the second year of life, childhood (4–7 years), and adolescence (9–15 years), to provide long-term protection against tetanus in all ages [3]. For previously non-immunized adolescents and adults, a total of 5 TTCV doses are recommended [3]. In countries where maternal and neonatal tetanus (MNT) is still a problem, pregnant women whose tetanus vaccination history is unknown or inadequate are recommended to receive 2 TTCV doses during their first pregnancy, with the first dose provided at first contact with health services and the second dose provided at least 4 weeks later and at least 2 weeks before delivery [3]. The third dose can be given at least 6 months after the second, and 2 boosters are provided during subsequent pregnancies or at least 1 year later. This schedule has been shown to reduce neonatal mortality from tetanus by 94% [5].

In industrialized countries, MNT was generally eliminated before vaccine introduction through clean birth practices [6]. Deliveries assisted by skilled birth attendants (SBA), who can ensure clean delivery practices and education on hygienic umbilical cord practices, has been shown to be an effective intervention to prevent MNT and to reduce neonatal mortality due to tetanus by 50–70% [6-9].

In 1988, NT was estimated to cause over 787,000 deaths per year [10]. To address the global burden of NT, in 1989, The World Health Assembly called for the elimination of NT by 1995, defined as an incidence of <1 NT case per 1,000 live births in every district annually [11]. Maternal tetanus elimination was added in 1999, and the goal was relaunched through a partnership with World Health Organization (WHO), United Nations Children’s Fund (UNICEF), and United Nations Population Fund (UNFPA) as the Maternal and Neonatal Tetanus Elimination (MNTE) Initiative, focusing on 59 priority countries (including the addition of Timor Leste and South Sudan) that had not yet achieved elimination as of 2000 [7, 12]. The strategies recommended by the UN agency partnership to achieve MNTE are 1) routine immunization of women during pregnancy with TTCV, 2) immunization of all women of reproductive age (WRA) living in high risk areas with 3 doses of TTCV, 3) delivery by SBA to ensure clean delivery practices, and 4) surveillance for NT cases [7]. High-risk districts for MNT are defined as those reporting clean delivery coverage of < 70% and coverage with at least 2 TTCV doses <80% during the past 5 years among pregnant women or WRA [12]. All WRA living in high-risk areas are provided 3 doses of TTCV irrespective of their previous vaccination status, through appropriately spaced supplementary immunization activity (SIA) rounds [12]. As a result of MNTE efforts, the estimated number of NT deaths has been reduced by 94% (49,000 estimated deaths) in 2013, compared to 1988 [13]. However, the target date for MNTE has been repeatedly missed and extended; the most recent target date was 2015 [7]. As of October 2016, 41 (69%) of the 59 priority countries identified have achieved MNTE [14].

Beyond MNTE, a goal of tetanus vaccination is to prevent tetanus in all ages [3].The Global Vaccine Action Plan (GVAP) set a target of 90% coverage with three doses of diphtheria, tetanus, and pertussis containing vaccine (DTP3/Penta) among children aged <12 months nationally, and 80% in every district by 2015 [15]. GVAP objectives also emphasize equitable provision of vaccines to underserved areas and marginalized groups, as well as introducing strategies to provide vaccinations to all ages (“life course approach”). High vaccination coverage and provision of tetanus booster doses throughout the lifespan would raise population immunity and sustain achievements of the MNTE Initiative.

The World Health Organization (WHO) African Region (AFR) consists of 47 countries in Sub-Saharan Africa that carry a substantial burden of the remaining MNT cases. In this paper, we describe the current status of progress towards MNTE in AFR, discuss the challenges and barriers to reaching elimination, and make recommendations for reaching and maintaining elimination in the region.

## Methods

For each country in AFR, we reviewed national vaccination schedules, vaccination coverage estimates and reported NT cases from the WHO-UNICEF joint reporting form available on the WHO website [16-21]. WHO-UNICEF coverage estimates were reviewed to determine coverage of the third dose of Diphtheria Tetanus and Pertussis vaccine (DTP3)/Pentavalent vaccine (Penta 3) among children aged <12 months and the proportion of births in a given year that can be considered as having been protected against tetanus (protected at birth, PAB) as a result of maternal immunization [19, 20]. WHO-UNICEF estimates of PAB were calculated on the basis of mathematical modeling [22], whereas PAB is usually defined as having received 2 tetanus toxoid (TT) doses during the last birth, ≥2 TT doses with the last dose ≥ 3 years prior to the last birth, ≥3 doses with the last dose ≤ 5 years prior, ≥4 doses with the last dose ≤10 years prior, or ≥ 5 prior doses [23]. Official country administrative coverage figures reported to WHO-UNICEF were reviewed for the proportion of women who have received their second or subsequent dose of TTCV during pregnancy (TT2+) [21]. Administrative TT2+ coverage is calculated by dividing the number of pregnant women who received the second or subsequent dose of TT by the estimated number of live births for the year [24]. 2005, 2010, and 2011-2015 averages forDTP3/Penta3, TT2+, and PAB coverage were calculated for countries yet to achieve MNTE and those that have achieved MNTE using the formula: (sum of (coverage x target population)) / sum of target populations). NT incidence rates were calculated for each country based on the number of reported cases divided by the number of births. Population denominators are from the United Nations Population Division [25].

The number of WRA (generally 15–49 years of age) targeted and reached with TT2+ by SIAs for each country yet to achieve elimination were reviewed from the WHO data on MNTE SIAs [26]. The proportion of deliveries attended by a SBA as reported to UNICEF were reviewed for each country in the region [27]. We searched PubMed and the WHO website for relevant articles and reports relating to MNTE globally or within AFR published up to September 1, 2016 [28]. Additionally, we reviewed presentations and reports from the Strategic Advisory Group of Experts Working Group on Maternal and Neonatal Tetanus Elimination and Broader Tetanus Prevention [29].

## Current status of knowledge

### Current status of MNTE Initiative in AFR

**Figure 1:**
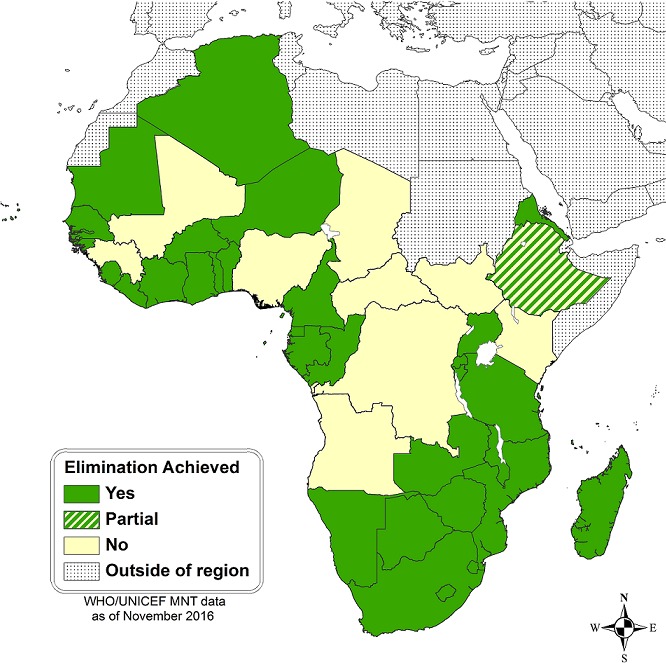
Maternal Neonatal Tetanus Elimination Status- WHO African Region, 2016

When the MNTE Initiative was re-launched in 1999, only 9 (19%) of the 47 countries in the WHO African Region had eliminated MNT (Algeria, Botswana, Cabo Verde, The Gambia, Lesotho, Mauritius, Sao Tome and Principe, Seychelles, and Swaziland). As of November 2016, 28 additional countries in the region have eliminated MNT, for a total of 37 (79%) countries, leaving 10 countries yet to achieve MNTE (Angola, Central African Republic (CAR), Chad, Democratic Republic of the Congo (DRC), Ethiopia (Somali Region only), Guinea, Kenya, Mali, Nigeria, South Sudan) (Figure 1). In those 10 countries, the risk of MNT is mainly limited to a few high-risk districts except in countries that are challenged with civil unrest and insecurity (CAR, DRC, Mali, and Nigeria).

### Tetanus vaccination coverage

Immunization programs of all 47 AFR countries recommend three TTCV doses for infants and five appropriately spaced doses for pregnant women and/or WRA, where appropriate [16]. Of the 37 AFR countries that have achieved MNTE, five (14%) include the three WHO-recommended TTCV booster doses for children older than 1 year of age in their immunization schedules (Algeria, Botswana, Mauritius, Seychelles, and South Africa); none of the 10 countries yet to achieve MNTE provide the three WHO-recommended tetanus booster doses in their vaccination schedule [16].

**Figure 2:**
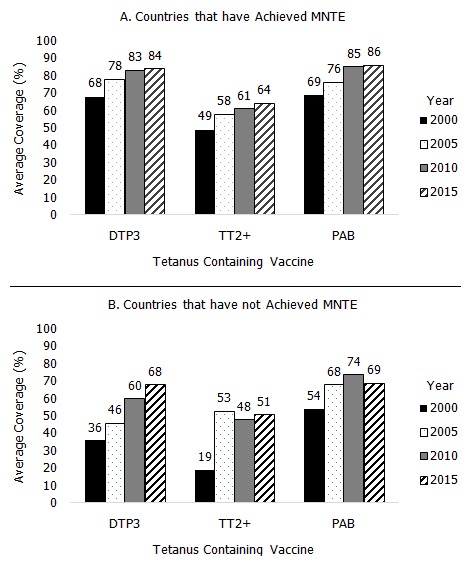
Average tetanus vaccination coverage of WHO African region countries by maternal and neonatal tetanus elimination status during 2000, 2005, 2010 and 2015

The regional average for DTP3/Penta3 coverage has increased from 52% in 2000 to 76% in 2015 overall, though coverage in several countries has not improved during this time period [19, 30]. In 2015, DTP3/Penta3 coverage among countries that had achieved MNTE was 84% compared with 68% among countries that had not yet achieved MNTE (Figure 2). Of the AFR countries that have achieved elimination, 16 (43%) had ≥90% DTP3/Penta3 coverage in 2015 (Table 1). Of the 10 countries that have not achieved MNTE, Kenya is the only country that has ever reached ≥90% DTP3/Penta3 coverage though not in every year since 2010 (Table 2). Ebola-affected countries (Guinea, Liberia, and Sierra Leone) experienced decreases in DTP3/Penta3 coverage rates during and following the outbreak in 2014 and 2015. Over the same time period, Angola and South Sudan had decreases in DTP3/Penta3 coverage, likely due to insecurity. Notably, DTP3/Penta3 coverage has been consistently low (<65%) in Equatorial Guinea which achieved elimination in 2016; such coverage may prove a challenge for maintaining elimination.

**Table 1:** WHO-UNICEF Coverage Estimates for the third dose of Diphtheria, Tetanus, and Pertussis Vaccine (DTP3/Penta3) among children aged <12 Months in countries that have achieved Maternal Neonatal Tetanus Elimination — WHO Africa Region 2000, 2005, and 2010-2015^a^

Country	2000	2005	2010	2011	2012	2013	2014	2015
Algeria^b^	86	88	95	95	95	95	95	95
Benin	78	70	76	75	81	74	75	79
Botswana^b^	97	96	95	95	95	95	95	95
Burkina Faso	45	82	91	91	90	88	91	91
Burundi	80	87	96	96	96	96	95	94
Cabo Verde^b^	90	95	99	90	94	93	95	93
Cameroon	62	80	84	82	85	89	87	84
Comoros	70	68	74	83	86	83	80	80
Congo	33	62	74	80	79	85	90	80
Cote d'Ivoire	65	76	85	62	82	80	76	83
Equatorial Guinea	34	39	44	41	24	3	20	16
Eritrea	81	96	90	96	94	94	94	95
Gabon	45	45	67	75	82	79	70	80
Gambia^b^	80	95	97	96	98	97	96	97
Ghana	88	84	94	91	92	90	98	88
Guinea-Bissau	49	68	80	80	80	80	80	80
Lesotho^b^	83	89	93	96	95	93	93	93
Liberia	46	60	70	77	80	76	50	52
Madagascar	57	85	70	73	70	74	73	69
Malawi	75	93	93	97	96	89	91	88
Mauritania	51	71	64	75	80	80	84	73
Mauritius^b^	88	97	99	98	98	98	97	97
Mozambique	70	80	74	76	76	78	79	80
Namibia	79	86	83	82	84	89	88	92
Niger	34	45	70	75	71	67	68	65
Rwanda	90	95	97	97	98	98	98	98
Sao Tome and Principe^b^	82	97	98	96	96	97	95	96
Senegal	52	84	89	92	91	92	89	89
Seychelles^b^	98	99	99	99	98	98	99	97
Sierra Leone	44	65	86	89	91	92	83	86
South Africa	73	72	66	72	68	65	70	69
Swaziland^b^	84	86	89	91	95	98	98	90
Togo	64	82	83	85	84	84	87	88
Uganda	52	64	80	82	78	78	78	78
United Republic of Tanzania	79	90	91	90	92	91	97	98
Zambia	85	82	83	81	78	79	86	90
Zimbabwe	78	68	89	93	95	95	91	87
Average^c^	68	78	83	84	84	84	85	84

a WHO-UNICEF estimates of DTP3 coverage. Last updated July 15, 2016. Available from http://apps.who.int/immunization_monitoring/globalsummary/timeseries/tswucoveragedtp3.html Accessed September 18, 2016.

b Eliminated Maternal and Neonatal Tetanus Prior to 2000.

c Average calculated as [sum of (coverage x surviving infants)] / sum of surviving infants. Denominator of total surviving infants to age 1 available from United Nations, Department of Economic and Social Affairs, Population Division (2015). World Population Prospects: The 2015 Revision, DVD Edition. Available from https://esa.un.org/unpd/wpp/Download/Standard/Population/ Accessed on September 18, 2016

**Table 2:** WHO-UNICEF coverage estimates for the third Dose of Diphtheria, Tetanus, and Pertussis Vaccine (DTP3/Penta3) among children Aged <12 months in countries that have not achieved Maternal Neonatal Tetanus Elimination — WHO Africa Region 2000, 2005, and 2010-2015^a^

Country	2000	2005	2010	2011	2012	2013	2014	2015
Angola	28	38	77	71	75	77	64	64
Central African Republic	37	54	45	47	47	23	47	47
Chad	36	25	39	33	45	48	46	55
DR of Congo	40	60	60	74	75	74	80	81
Ethiopia	30	44	61	65	69	72	77	86
Guinea	46	59	64	63	62	63	51	51
Kenya	82	76	90	96	94	87	92	89
Mali	43	77	73	66	68	71	77	68
Nigeria	29	36	54	48	42	46	49	56
South Sudan	NA	NA	NA	61	59	45	39	31
Average^b^	36	46	60	62	61	62	65	68

a WHO-UNICEF estimates of DTP3 coverage. Last updated July 15, 2016. Available from http://apps.who.int/immunization_monitoring/globalsummary/timeseries/tswucoveragedtp3.html Accessed September 18, 2016.

b Average calculated as [sum of (coverage x surviving infants)] / sum of surviving infants. Denominator of total surviving infants to age 1 available from United Nations, Department of Economic and Social Affairs, Population Division (2015). World Population Prospects: The 2015 Revision, DVD Edition. Available from https://esa.un.org/unpd/wpp/Download/Standard/Population/ Accessed on September 18, 2016.

Both TT2+ and PAB coverage are estimates of tetanus immunity among pregnant women. The percentage of pregnant women covered with TT2+ among all countries in the AFR region increased from 44% in 2000 to 69% in 2015; PAB increased from 62% to 77% during the same time period [30]. In countries that have achieved MNTE, the average PAB and TT2+ coverage for 2015 was 86% and 64%, respectively (Figure 2), with ≥ 80% TT2+ or PAB coverage in all but Equatorial Guinea, Madagascar, and Zimbabwe (Table 3). Of the AFR countries that have not yet achieved MNTE, the average PAB and TT2+ coverage in 2015 was 69% and 64%, respectively (Figure 2); among these countries, ≥80% TT2+ or PAB coverage was achieved in Chad, DRC, Ethiopia, Guinea, Kenya, and Mali (Table 4). Guinea and Liberia, which were affected by the 2014 Ebola outbreak, had lower TT2+ coverage in 2014 and 2015 relative to other years, but PAB remained stable. However, Sierra Leone, also affected by the Ebola outbreak, did not have a drop in TT2+ or PAB coverage.TT2+ coverage can underestimate true protection from tetanus, especially in countries with well-established vaccination programs (e.g. Botswana, Cabo Verde, and Comoros), because it excludes women unvaccinated during pregnancy but already protected through previous vaccination or who received one dose in pregnancy and had undocumented previous doses (Table 3) [23, 24].

**Table 3:** Tetanus coverage^a^,^b^ among pregnant women in countries that have not achieved Maternal and Neonatal Tetanus Elimination — WHO African Region, 2000, 2005, and 2010-2015

	2000		2005		2010		2011		2012		2013		2014		2015	
Country	PAB	TT 2-Ι-	PAB	TT 2-Ι-	PAB	TT2+	PAB	TT2+	PAB	TT2+	PAB	TT2+	PAB	TT2+	PAB	TT2+
Alqeria^c^	64	ΝΑ	69	ΝΑ	90	NA	90	NA	90	NA	90	NA	92	NA	92	NA
Benin	87	81	95	65	92	63	92	77	93	82	93	69	93	69	85	77
Botswana^c^	80	45	83	72	92	64	92	81	92	79	92	70	92	52	92	53
Burkina Faso	57	NA	72	71	85	82	88	82	88	86	88	71	89	91	92	91
Burundi	51	28	65	33	94	94	80	99	85	88	85	96	85	80	85	90
Cabo Verde^c^	60	45	70	55	92	NA	92	NA	92	64	92	63	92	82	92	64
Cameroon	54	40	76	60	91	74	75	69	85	65	85	68	85	64	85	62
Comoros	57	40	91	80	85	38	85	65	85	61	85	NA	85	51	85	55
Congo	67	39	75	62	83	92	83	92	83	85	83	85	85	90	85	85
Cote d'Ivoire	76	78	75	34	82	62	82	59	82	84	82	81	82	77	85	80
Equatorial Guinea	61	30	59	25	75	54	75	64	75	30	75	31	70	36	70	23
Eritrea	80	25	84	70	93	35	93	35	94	35	94	94	94	35	94	65
Gabon	39	16	58	34	75	51	75	57	75	59	85	57	85	54	85	59
Gambia^c^	92	90	89	71	91	88	91	61	92	71	82	78	92	60	92	82
Ghana	69	73	83	71	86	80	88	76	88	72	88	71	88	42	88	78
Guinea-Bissau	49	NA	65	40	78	70	80	32	80	45	80	38	80	70	80	83
Lesotho^c^	73	NA	79	NA	83	80	83	57	83	36	83	39	83	47	83	68
Liberia	51	25	60	72	91	63	91	74	91	74	91	76	89	68	89	63
Madagascar	58	40	66	47	76	58	78	62	78	59	78	63	78	53	78	47
Malawi	84	61	86	63	87	89	87	79	89	71	89	65	89	90	89	54
Mauritania	44	NA	59	34	87	30	80	29	80	40	80	40	80	42	80	37
Mauritius^c^	79	76	86	88	95	96	95	95	95	95	95	85	95	85	95	83
Mozambique	75	61	77	62	83	69	83	70	83	70	83	64	83	66	83	80
Namibia	74	60	80	99	83	75	83	64	83	67	83	72	85	71	85	70
Niger	63	31	69	54	84	85	84	93	84	83	81	89	81	90	81	73
Rwanda	81	NA	79	54	85	58	85	62	85	76	85	78	90	92	90	99
Sao Tome and Principes	NA	75	99	99	99	87	99	90	99	92	99	90	99	91	99	92
Senegal	62	45	80	67	88	60	88	NA	91	70	91	88	91	84	91	84
Seychelles^c^	NA	NA	NA	99	NA	99	NA	99	NA	NA	NA	99	NA	97	NA	99
Sierra Leone	53	20	83	66	85	90	85	90	87	60	87	81	85	90	85	92
South Africa	68	65	59	43	77	NA	77	51	77	63	77	NA	80	NA	80	NA
Swaziland^c^	80	94	82	83	86	90	86	79	86	53	86	72	88	72	88	57
Togo	63	47	81	70	81	85	81	86	81	78	77	78	81	80	81	78
Uganda	70	42	85	55	85	53	85	49	85	49	85	56	85	56	85	58
United Republic of Tanzania	79	77	81	81	83	73	88	76	88	79	88	79	88	93	90	94
Zambia	78	61	91	83	90	72	81	74	81	71	81	74	85	74	85	75
Zimbabwe	76	60	77	80	76	60	66	80	66	85	66	75	75	58	75	NA
Averaqe^d^	69	49	76	58	85	61	84	64	85	67	84	61	85	64	86	64

a PAB=Protection at birth. PAB is usually defined as receiving 2 tetanus toxoid containing vaccine (TTCV) doses during the last birth, >2 TTCV doses with the last dose <3 years prior to the last birth, >3 doses with the last dose <5 years prior, >4 doses with the last dose <10 years prior, or >5 prior doses. WHO-UNICEF annual estimates of PAB were calculated on the basis of mathematical modeling WHO-UNICEF estimates of PAB coverage. Last updated July 15, 2016. Available from http://apps.who.int/immunization_monitoring/globalsummary/timeseries/tswucoveragepab.html Accessed December 18, 2016.

b TT2+ = Proportion of women who have received their second or subsequent dose of TTCV during pregnancy. Reported estimates of TT2+ coverage. Last updated November 18, 2016. Available from http://apps.who.int/immunization_monitoring/globalsummary/timeseries/tscoveragett2plus.html Accessed December 18, 2016.

c Eliminated Maternal and Neonatal Tetanus Prior to 2000.

d Average calculated as [sum of (coverage χ births)] / sum of births. Denominator of births available from United Nations, Department of Economic and Social Affairs, Population Division (2015). World Population Prospects: The 2015 Revision, DVD Edition. Available from https://esa.un.org/unpd/wpp/Download/Standard/Population/Accessed on September 18, 2016.

**Table 4:** Tetanus coverage^a^,^b^ among pregnant women in countries that have not achieved Maternal and Neonatal Tetanus Elimination — WHO African Region, 2000, 2005, and 2010-2015

	2000		2005		2010		2011		2012		2013		2014		2015	
Country	PAB	TT2+	PAB	TT2+	PAB	TT2+	PAB	TT2+	PAB	TT2+	PAB	TT2+	PAB	TT2+	PAB	TT2+
Angola	60	NA	75	53	75	87	70	79	72	78	75	83	78	76	78	71
Central African	36	20	48	34	86	50	80	81	66	76	66	33	60	53	60	52
Republic																
Chad	39	12	61	NA	60	77	60	60	43	70	50	78	60	83	75	96
DR of Congo	45	25	67	66	77	85	70	84	70	86	75	85	82	88	82	91
Ethiopia	54	32	80	45	88	NA	88	1	68	66	72	NA	80	NA	80	92
Guinea	79	43	90	75	90	70	80	80	80	86	80	93	80	62	80	54
Kenya	68	51	73	72	78	72	73	76	73	NA	73	77	76	55	80	55
Mali	50	62	86	63	85	59	89	60	89	66	85	95	85	86	85	63
Nigeria	57	NA	62	52	69	39	60	44	60	56	60	39	55	44	55	40
South Sudan	NA	NA	NA	NA	NA	NA	NA	61	NA	50	NA	41	NA	45	NA	28
Average^c^	54	19	68	53	74	48	69	51	65	61	66	51	68	50	69	64

a PAB=Protection at birth. PAB is usually defined as receiving 2 tetanus toxoid containing vaccine (TTCV) doses during the last birth, >2 TTCV doses with the last dose <3 years prior to the last birth, >3 doses with the last dose <5 years prior, >4 doses with the last dose <10 years prior, or >5 prior doses. WHO-UNICEF annual estimates of PAB were calculated on the basis of mathematical modeling WHO-UNICEF estimates of PAB coverage. Last updated July 15, 2016. Available from http://apps.who.int/immunization_monitoring/globalsummary/timeseries/tswucoveragepab.html Accessed December 18, 2016.

b TT2+ = Proportion of women who have received their second or subsequent dose of TTCV during pregnancy. Reported estimates of TT2+ coverage. Last updated November 18, 2016. Available from http://apps.who.int/immunization_monitoring/globalsummary/timeseries/tscoveragett2plus.html Accessed December 18, 2016.

c Average calculated as [sum of (coverage χ births)] / sum of births. Denominator of births available from United Nations, Department of Economic and Social Affairs, Population Division (2015). World Population Prospects: The 2015 Revision, DVD Edition. Available from https://esa.un.org/unpd/wpp/Download/Standard/Population/ Accessed on September 18, 2016.

During 1999 to the end of 2016, over 79 million WRA were vaccinated with at least 2 TTCV doses during SIAs in AFR countries, but by the end of 2016 about 5.5 million WRA in AFR countries still need to be reached. Only a limited number of districts are assessed as high-risk in the 10 countries remaining to achieve MNTE (e.g., in DRC only 11 of the 514 health zones are high-risk). The total number of TTSIA phases completed as of April 2016 and number of TT2+ doses provided through SIAs are reported for countries yet to achieve MNTE in Table 5. Most of the required funds are available to support Angola, Chad, DRC, Ethiopia, Guinea, Kenya, and South Sudan to complete all their planned and final TTCV SIAs; these countries are on course to achieve elimination by 2018 [31]. However, most of the funding for TTCV SIAs in CAR, Mali, and Nigeria is still lacking [31].

**Table 5:** Number of TT supplementary immunization activities and years conducted, number of TT2+ doses provided through SIAs, current skilled birth attendant coverage, and reported neonatal tetanus cases and incidence in countries that have not achieved Maternal and Neonatal Tetanus Elimination - African Region

Country	No. of TT SIA phases^a^ (years conducted)	No. of TT2+ doses providedthrough SIAs^b^	No. of WRA (aged 15-49 years) in 2015^c^	% births attended by SBA^d^(year measured)	No. ofreported NT cases in 2015^e^	Reported incidence of NT cases in 2015 per 1,000 births^f^,^g^
Angola	4 Phases (2003-2014)	7,097,552	5,570,000	47 (2007)	36	0.03
CAR	1 phase (2008)	804,984	1,225,000	54 (2010)	96	0.59
Chad	5 phases (2000-2016)	3,045,147	3,091,000	24 (2015)	195	0.31
DRC	8 phases (2004-2014)	10,293,095	17,320,000	80 (2014)	330	0.10
Ethiopia	13 phases(19992016)	12,786,464^h^	24,103,000	16 (2014)	18	0.01
Guinea	4 phases(2003-2012)	3,140,970	2,932,000	45 (2012)	NA	NA
Kenya	6 phases(2002-2016)	4,339,702	11,175,000	62 (2014)	32	0.02
Mali	8 phases(2002-2014)	3,802,724	3,846,000	56 (2006)	8	0.01
Nigeria	4 phases(2009-2016)	2,488,283	41,363,000	38 (2013)	53	0.01
South Sudan	12 phases (20012016)	3,226,433	2,935,000	19 (2010)	0	0

Abbreviations: CAR=Central African Republic; DRC=Democratic Republic of Congo; SIAs=supplemental immunization; SBA=skilled birth attendant; NA=not available; NT=neonatal tetanus

a In most cases, phase refers to 3 rounds of SIAs in particular set of high-risk districts

b Supplementary Immunization Activities MNTE. Last updated April 21, 2016. Available at http://www.who.int/immunization/diseases/MNTE_initiative/en/index7.html Accessed December 19, 2016. Women may be targeted and reached more than once through SIAs.

c Population of females aged 15-49 years from United Nations, Department of Economic and Social Affairs, Population Division (2015). World Population Prospects: The 2015 Revision, DVD Edition. Available from https://esa.un.org/unpd/wpp/Download/Standard/Population/ Accessed on December 18, 2016.

d UNICEF global databases 2016 based on DHS, MICS and other nationally representative surveys. Last updated February, 2016. Available at http://data.unicef.org/maternal-health/delivery-care.html Accessed September 18, 2016.

e Tetanus (neonatal) reported cases. Last updated December 1, 2016. Available at http://apps.who.int/immunization_monitoring/globalsummary/timeseries/tsincidencentetanus.html Accessed December 18, 2016.

f Denominator data of number of births Available from United Nations, Department of Economic and Social Affairs, Population Division (2015). World Population Prospects: The 2015 Revision, DVD Edition. Available from https://esa.un.org/unpd/wpp/Download/Standard/Population/ Accessed on December 18, 2016.

g Reported NT incidences are national and do not represent district level data used to determine elimination status (<1 NT case per 1000 live births in every district annually). Underreporting of NT cases is common, with the efficiency of notification for NT cases globally estimated at less than 11% by Khan et al [2].

h TT2+ doses are missing for phases 11 and 12 because round-wise coverage was reported instead of individual level coverage for receipt of multiple doses.

### Clean delivery and cord care

In addition to vaccination, clean delivery and cord care are essential interventions to prevent and eliminate MNT. The proportion of births delivered by SBAs is a key indicator for monitoring progress towards MNTE. In AFR, the percentage of births attended by SBAs was 54% in 2013 [32]. Of countries that have achieved MNTE, 17 of 37 (46%) have >70% SBA coverage, while 20 (54%) have <70% SBA coverage; Eritrea (34% in 2010), Niger (40% in 2015), Madagascar (44% in 2013), Guinea-Bissau (45% in 2014), and United Republic of Tanzania (49% in 2014) have SBA coverage <50% [27]. Four of these countries with <50% SBA coverage have >80% PAB coverage, while Madagascar has 78% PAB coverage. All countries that have not achieved MNTE have <70% (range 19%-62%) SBA coverage in their last national survey except for DRC, which achieved 80% SBA coverage nationally in 2014 (Table 5). Continued low PAB and SBA coverage could be a risk for sustaining MNTE.

### Surveillance

Sensitive NT surveillance is important for conducting epidemiologic analysis of cases, planning targeted interventions, and evaluating program effectiveness. WHO recommends routine monthly surveillance of NT, including zero reporting, active surveillance, and retrospective record review at major health facilities at least once a year to identify previously unreported NT cases [33]. During 2015, the number of NT cases reported from countries that have not achieved MNTE ranged from 0 in South Sudan to 330 in the DRC, with a respective reported national incidence of 0 to 0.59 NT cases per 1,000 live births (Table 5).However, underreporting of NT surveillance is common, with the efficiency of notification for NT cases globally estimated at less than 11% [2]. Likely causes of NT underreporting include the general occurrence of NT cases in remote and underserved areas, NT deaths occurring in the community without being seen by a health worker, and lack of a robust system of active NT surveillance in most countries.

Serosurveys can help monitor progress towards achieving or maintaining MNTE, especially in countries or areas where TT2+ or PAB coverage may not be accurate. In Burundi in 1989, a national serosurvey among women giving birth in the previous year reported 73% PAB coverage and 67% seroprotection [34]. More recently, a national serosurvey in CAR in 1995 reported 74% PAB and 89% seroprotection among women giving birth in the past year [35]. In Cambodia in 2012, seroprotection was 88% for WRA overall, while parous women had 83% PAB and 97% seroprotection [23]. PAB coverage can be underestimated due to residual immunity from infant doses in some adult women, booster doses provided outside routine services, misclassification of PAB status due to poor availability of documentation, and recall bias [23]. Serosurveys can help estimate population immunity, which contributes to validation of MNTE, and identify areas at high risk for MNT [36]. Integration of tetanus testing in serosurveys for other antigens (e.g., rubella) using multiplex testing may reduce the cost and resources required [23].

### Challenges and recommendations for AFR countries that have not yet achieved MNTE

A number of challenges exist among the countries that have not yet achieved MNTE. In all the countries that have not yet achieved elimination, weak health systems affect their ability to effectively implement the four MNTE strategies. In most countries, sub-optimal routine immunization is evidenced by low rates of DTP3/Penta3 and TT2+/PAB coverage. The proportion of safe deliveries occurring in health facilities or at home with skilled birth attendants also remains low. Insecurity and unrest in CAR, Nigeria, Mali, and some areas of other countries result in WRAs living in high-risk areas being inaccessible. These countries also have challenges with funding the remaining TTCV SIAs needed to achieve MNTE. Vaccine controversy issues related to targeted vaccination of WRA have also occurred, resulting in varying extents of vaccine hesitancy. In Kenya, the national Conference of Catholic Bishops made public allegations that tetanus vaccine could cause sterility in women, which has delayed the country’s timeline for MNTE [37]. Furthermore, AFR countries face competing priorities both within the EPI program, such as polio eradication (e.g., Nigeria) and measles elimination activities (i.e., throughout AFR), response campaigns to meningitis serogroup A and yellow fever outbreaks, and with other health programs (e.g., Ebola, HIV, TB, malaria).

Despite these challenges, MNTE is achievable in the remaining 10 AFR countries if there is strong national commitment, timely availability of resources, high quality micro plans, well implemented activities with monitoring and supervision, active community engagement, and adequate coordination with and implementation of maternal and child health services [31]. The methods to approach the MNTE strategies to reach elimination vary by country.

Efforts to strengthen the health care system of AFR countries yet to achieve MNTE could result in increased vaccination of pregnant women through routine services along with improving rates of clean deliveries. Eritrea, Rwanda, South Africa, and Zimbabwe were successful at eliminating MNT through efforts to strengthen the health system, without conducting any TTCV SIAs [31]. To achieve MNTE, Eritrea and Liberia promoted clean deliveries through additional training of traditional birth attendants [38, 39]. Liberia also encouraged facility births with distribution of free kits for mothers, including baby articles and bed nets [39]. Burundi constructed and staffed rural health facilities with trained health workers to provide free health care for mothers and children, in addition to conducting catch-up vaccination during mother and child health weeks [40]. Immunization programs should encourage using every opportunity to provide TTCV to women (e.g., child health days, outreach immunization sessions, periodic intensification of routine immunization, antenatal care visits, family planning visits) to improve coverage. Providing TTCV booster doses in the routine immunization schedule (e.g., in the second year of life with MCV2, or in school) can ensure women are protected, or need fewer booster doses to be protected, by the time they reach reproductive age. Mozambique provides TTCV to WRA during routine EPI sessions and also in secondary schools and workplaces; in addition, children of both sexes are provided TTCV in the first and second grade resulting in continued seroprotection of older children and adolescents [41, 42].

Many countries conduct multiple polio and measles SIAs, with many targeting hard-to-reach, inaccessible or previously inaccessible areas (i.e., conflict affected). Integrated campaigns that include TTCV could be an opportunity to reach WRA in high-risk areas. For countries with inaccessible areas or insecurity, innovative approaches such as use of TT-Uniject™ (a heat-stable prefilled injection device) could help reach these populations and improve TTCV coverage for WRA. An evaluation in Mali, where there are a limited number of health workers, showed that community-based volunteers could safely administer TT-Uniject™, communities were accepting of the practice, and previously inaccessible populations were reached [43]. Use of TT-Uniject™ during campaigns would allow unskilled vaccinators to provide vaccine, similar to polio SIAs.

India achieved MNTE in 2015 by employing a combination of approaches that may be applicable for some AFR countries. India faced similar challenges to the AFR region including a history of unsafe birth practices during home deliveries and suboptimal TTCV coverage [44]. Some of the approaches used in India to achieve MNTE included addition of TTCV booster doses to the childhood immunization schedule, immunization of pregnant women during antenatal care visits, implementation of SIAs in high-risk areas, and extension of outreach services to include vaccination of children, adolescent, and pregnant women with TTCV. India also promoted institutional deliveries through the provision of conditional cash transfers, communication with communities on clean cord care practices, and distribution of clean delivery kits to SBAs [44, 45].

As mentioned above, sensitive NT surveillance is a challenge for countries trying to achieve MNTE. Developing standardized NT surveillance guidelines with performance indicators similar to those used for acute flaccid paralysis (AFP for polio) and measles-rubella (MR) surveillance are needed to improve NT surveillance; such guidelines and indictors already exist for the Region of the Americas and could be adapted to other regions. Active surveillance for NT cases has been successfully integrated into existing active case-based surveillance activities for AFP and MR in many countries, resulting in more robust NT reporting. For example in Nepal, NT surveillance was integrated into the AFP surveillance system, which uses a network of surveillance medical officers reporting weekly from 413 sentinel surveillance sites (10% of government health facilities), and investigating suspected NT cases using a standard case-investigation form [46]. Since many neonatal deaths occur at home, it is important to supplement health facility-based surveillance with community based surveillance. In addition, sensitivity of NT surveillance may be improved through investigation of neonatal deaths reported through vital registration and reporting [31].

### Sustaining elimination

Because tetanus cannot be eradicated from the environment, countries need to have a plan for sustaining MNTE. Attention to MNT will likely decrease after elimination along with resources. However, efforts to ensure clean deliveries, maintain high immunization coverage of pregnant women, and strengthen NT surveillance should be continued through integration with routine programs. Countries with weak health systems that achieved elimination through SIAs need to have employed additional strategies for reaching women beyond routine services, which may include continued SIAs in high risk areas and outreach activities during child health days or antenatal care, to ensure population immunity does not lapse after MNTE validation [7].

Providing TTCV booster doses during the second year of life, childhood, and adolescence in the routine schedule will help ensure individuals of all ages and both sexes are adequately protected without relying on SIAs [3]. As school attendance has increased in the African region, school-based immunizations platforms are increasingly attractive platforms for administering booster doses during childhood and adolescence. In 2012, 24% of AFR countries reported providing vaccinations in school [47]. Opportunities to provide TTCV boosters increasingly exist as AFR countries, many with funding assistance from GAVI, the Vaccine Alliance, introduce vaccinations given beyond the first year of life, including a second dose of measles containing vaccine (MCV2) in the second year of life (2YL) and human papillomavirus vaccine in early adolescence [48]. As of September 2016, 25 (53%) AFR countries provided MCV2, most during the 2YL [16]. Additionally, booster doses would address tetanus immunity gaps in older children and adult men in the region [42, 49]. Attention was brought to the seriousness of this issue after cases of tetanus were reported following voluntary medical male circumcision in Eastern and Southern African countries [49, 50]. When countries develop plans to maintain MNTE, it is important to include booster doses for both sexes to improve equity, ensure long-term protection and decrease the burden of tetanus in all ages.

Most countries in the African meningitis belt have or will introduce the meningococcal serogroup A polysaccharide TT-conjugate vaccine (MenAfriVac) by conducting SIAs targeting persons aged 1–29 years, followed by introduction into the routine immunization schedule during the first or second year of life [16]. Results from a serosurvey conducted in Mali before and after the MenAfriVac campaign showed significant increases in tetanus immunity 2 years after the SIA [51]. A 25% reduction in NT cases was observed after MenAfriVac SIAs in 5 African countries, compared with before the SIA [52]. Therefore, MenAfriVac introduction in AFR countries in the meningitis belt could help boost tetanus immunity in those populations and support MNTE.

Effective NT surveillance, including sensitive and timely identification of NT cases, targeted implementation of control strategies, and effective monitoring and evaluation of the impact of these strategies, will be critical to sustaining MNTE. In addition to improving NT surveillance as mentioned above, annual reviews of data should be done to identify districts that could be at high-risk for MNT resurgence and need to implement additional control strategies to sustain MNTE [31]. In countries that have achieved MNTE, tetanus serosurveys of tetanus immunity in countries that have achieved MNTE can help supplement district level data reviews by identifying immunity gaps, which can provide evidence for the need for booster doses, including optimal schedules, and the need for targeted vaccination [31, 36].

## Conclusion

Though MNTE was not achieved in AFR by the target date of 2015, significant progress has been made in the region where 37 (79%) of 47 countries have achieved elimination as of November 2016. Table 6 summarizes the key findings of the MNTE strategies in the AFR region. In 2015, PAB and TT2+ coverage increased to 77% and 69% compared to 62% and 44% in 2000, respectively. Nevertheless, the region has 10 (56%) of the 18 countries worldwide that are yet to achieve MNTE. The majority of these countries are challenged with insecurity, weak health systems, competing priorities, and/or insufficient funding. In countries with insecurity, use of pre-filled injection devices, and integration of TTCV SIAs with polio and/or measles vaccination SIAs, or post-conflict campaigns, could help ensure tetanus protection among WRA in high-risk areas. To achieve and maintain MNTE, programs should use every available opportunity to provide TTCV to pregnant women, including in antenatal clinics, outreach, and during child health days. Providing the WHO-recommended TTCV booster doses to both sexes, e.g., in the 2YL, childhood (4-7 years), and adolescence (9–15 years), would help ensure women are protected before pregnancy and reduce the tetanus burden across the whole population. Case-based NT surveillance could be strengthened by development of standardized performance indicators and integration with active surveillance for AFP and MR. Countries that have achieved MNTE should develop plans to maintain elimination, including efforts to ensure high-quality NT surveillance and high tetanus immunity among WRA. Serosurveys may provide important information on tetanus population immunity for countries that have yet to achieve, as well as those that have already achieved MNTE.

**Table 6:** Summary of Current Key Findings on the Status of Maternal and Neonatal Tetanus Elimination (MNTE) Strategies in the WHO African Region

Programmatic areas	Key Findings
Tetanus Vaccination	Only 5/47 (11%) countries include the WHO-recommended 3 tetanus booster doses in their immunization schedules
	Regional average DTP3/Penta3 coverage has increased from 52% in 2000 to 76% in 2015
	Among countries that have achieved MNTE, average TT2+ coverage among pregnant women and PAB have increased from 49% and 69% in 2000 to 84% and 86% in 2015, respectively
	Among countries that have not achieved MNTE average TT2+ coverage among pregnant women and PAB have increased from 19% and 54% in 2000 to 64% and 69% in 2015, respectively
	By the end of 2016, over 79 million WRA still need to be reached with at least 2 TTCVs
Clean Delivery and Cord Care	In 2013, the average percentage of births attended by SBAs was 54%
	Among countries that have achieved MNTE, 17/37 (46%) have >70% SBA coverage
	Among countries that have not achieved MNTE, 1/10 (10%) have >70% SBA coverage
Neonatal Tetanus Surveillance	NT surveillance is weak in most countries leading to underreporting of cases, with the efficiency of notification for NT cases globally estimated at less than 11%
	NT surveillance could be strengthened with case investigations, standardized performance indicators, and better integration with active surveillance for measles and acute flaccid paralysis
	Serosurveys can help monitor progress towards achieving or maintaining MNTE

WHO=World Health Organization; MNTE= Maternal and Neonatal Tetanus Elimination; WRA = Women of Reproductive Age; TTCV = Tetanus Toxoid Containing Vaccines; SAB = Skilled Birth Attendants; NT= Neonatal Tetanus

Sources and calculations for averages are described in the methods section

### What is known about this topic

- Maternal and neonatal tetanus is preventable through appropriate vaccination of pregnant women and implementation of clean delivery and umbilical cord practices;
- Because of efforts towards maternal and neonatal tetanus elimination (MNTE), neonatal mortality from tetanus has declined an estimated 94% between 1988 and 2013;
- Worldwide, 41 (69%) of 59 priority countries have achieved MNTE, with 18 countries yet to achieve elimination, including 10 countries in the African region.

### What this study adds

- As of November 2016, over 79 millions women of reproductive age in countries in the African region (AFR) have been reached with at least 2 doses of tetanus-toxoid containing vaccines through supplemental immunization activities;
- Challenges to achieving MNTE in AFR countries include insecurity, weak health systems, competing priorities, and insufficient funding, but 7 of 10 AFR countries are on track to achieve MNTE by 2018;
- Only 5 (11%) AFR countries currently provide the 3 WHO-recommended tetanus booster doses during childhood, adolescence, and early adulthood to provide long-term protection against tetanus and assist with sustaining MNTE.

## Competing interests

The authors declare no competing interests.
